# Inactivation of wild-type p53 by a dominant negative mutant renders MCF-7 cells resistant to tubulin-binding agent cytotoxicity

**DOI:** 10.1054/bjoc.2001.2017

**Published:** 2001-09

**Authors:** C M Galmarini, N Falette, E Tabone, C Levrat, R Britten, N Voorzanger-Rousselot, O Roesch-Gateau, A Vanier-Viornery, A Puisieux, C Dumontet

**Affiliations:** INSERM 453, Laboratoire de Cytologie Analytique, Faculté de Médecine Rockefeller 8, Avenue Rockefeller, Lyon CEDEX 08, 69373, France; Service de Pathologie, Centre Léon Bérard, Lyon, 69008, France; INSERM 189-Faculté de Médecine Lyon-Sud BP 12, Oullins, 69921, France; Department Experimental Oncology, Cross Cancer Institute, Edmonton, Alberta T6G1Z2, Canada

**Keywords:** p53, drug resistance, paclitaxel, breast cancer cell lines, apoptosis

## Abstract

The present study was performed to gain insight into the role of p53 on the cytotoxicity of tubulin-binding agents (TBA) on cancer cells. Drug sensitivity, cell cycle distribution and drug-induced apoptosis were compared in 2 lines derived from the mammary adenocarcinoma MCF-7: the MN-1 cell line containing wild-type p53 (wt-p53) and the MDD2 line, containing a dominant negative variant of the p53 protein (mut-p53). The MDD2 cell line was significantly more resistant to the cytotoxic effects of vinblastine and paclitaxel than the MN1 cell line. MN1 cells, but not MDD2 cells, displayed wt-p53 protein accumulation as well as p21/WAF1 and cyclin G1 induction after exposure to TBA. Both cell lines arrested at G_2_/M after drug treatment. However exposure of MN1 cells to TBA resulted in a stronger variation in mitochondrial membrane potential, associated with cleavage of PARP, and more apoptosis, as measured by annexin V expression. After exposure to vinblastine, Raf 1 kinase activity was reduced in MDD2 cells but not in MN1 cells. Addition of flavopiridol to vinblastine- and paclitaxel-treated cells reversed the MDD2-resistant phenotype by inducing G_1_ cell cycle arrest and inhibiting endoreduplication. We conclude that the p53 status of cancer cells influences their sensitivity to TBA cytotoxicity. This effect is likely to involve differences in the apoptotic cascade. © 2001 Cancer Research Campaignhttp://www.bjcancer.com


*British Journal of Cancer* (2001) **85**(6), 902–908
© 2001 Cancer Research Campaign
doi: 10.1054/ bjoc.2001.2017, available online at http://www.idealibrary.com on IDEAL

http://www.bjcancer.com

### Inactivation of wild-type p53 by a dominant negative mutant renders MCF-7 cells resistant to tubulin-binding agent cytotoxicity

**CM Galmarini[1], N Falette[1], E Tabone[2], C Levrat[3], R Britten[4], N Voorzanger-Rousselot[1], O Roesch-Gateau[3], A Vanier-Viornery[1], A Puisieux[1] and C Dumontet[1]**

[1]INSERM 453, Laboratoire de Cytologie Analytique, Faculté de Médecine Rockefeller 8, Avenue Rockefeller, 69373, Lyon CEDEX 08, France; [2]Service de Pathologie, Centre Léon Bérard, 69008 Lyon, France; [3]INSERM 189-Faculté de Médecine Lyon-Sud BP 12 69921, Oullins, France; [4]Department Experimental Oncology, Cross Cancer Institute, Edmonton T6G1Z2, Alberta, Canada

**Summary** The present study was performed to gain insight into the role of p53 on the cytotoxicity of tubulin-binding agents (TBA) on cancer cells. Drug sensitivity, cell cycle distribution and drug-induced apoptosis were compared in 2 lines derived from the mammary adenocarcinoma MCF-7: the MN-1 cell line containing wild-type p53 (wt-p53) and the MDD2 line, containing a dominant negative variant of the p53 protein (mut-p53). The MDD2 cell line was significantly more resistant to the cytotoxic effects of vinblastine and paclitaxel than the MN1 cell line. MN1 cells, but not MDD2 cells, displayed wt-p53 protein accumulation as well as p21/WAF1 and cyclin G1 induction after exposure to TBA. Both cell lines arrested at $G_2$/M after drug treatment. However exposure of MN1 cells to TBA resulted in a stronger variation in mitochondrial membrane potential, associated with cleavage of PARP, and more apoptosis, as measured by annexin V expression. After exposure to vinblastine, Raf 1 kinase activity was reduced in MDD2 cells but not in MN1 cells. Addition of flavopiridol to vinblastine- and paclitaxel-treated cells reversed the MDD2-resistant phenotype by inducing $G_1$ cell cycle arrest and inhibiting endoreduplication. We conclude that the p53 status of cancer cells influences their sensitivity to TBA cytotoxicity. This effect is likely to involve differences in the apoptotic cascade. © 2001 Cancer Research Campaign http://www.bjcancer.com

**Keywords**: p53; drug resistance; paclitaxel; breast cancer cell lines; apoptosis

Tubulin-binding agents (TBA), currently represented in the clinic by vinca alkaloids and taxanes, are unique among antimitotic agents in that they do not target nucleic acids. Vinca alkaloids such as vinblastine can bind both to soluble tubulin dimers and microtubules and, at high concentrations, inhibit microtubule polymerization (Jordan et al, 1992; Lobert et al, 1996; Nagan et al, 2000). Taxanes, such as paclitaxel and the related compound docetaxel, only bind to polymerized tubulin and promote microtubule polymerization (Jordan et al, 1993; Liu et al, 1994). At clinically achievable concentrations, both vincas and taxanes induce apoptotic cell death by inhibiting microtubule dynamics, without altering the percentage of tubulin polymerization. It is generally accepted that TBA inhibit cell proliferation by inducing a sustained mitotic arrest at the metaphase/anaphase transition, which is associated with the formation of an incomplete metaphase plate of chromosomes and an abnormal assembly of spindle microtubules (Jordan et al, 1996; Rudner and Murray, 1996). However other mechanisms for taxane-induced cytotoxicity have been reported, including a late $G_1$ block in non-transformed cells (Trielli et al, 1996) and inhibition of centrosomal duplication in late $G_2$ (Paoletti et al, 1997).

*Received 7 February 2001*
*Revised 19 June 2001*
*Accepted 20 June 2001*

*Correspondence to:* CM Galmarini

The effect of p53 status on sensitivity of cancer cells to chemotherapeutic agents must take into account the dual role of p53 protein as a guardian of genome integrity and as a key intermediate of apoptosis (Lane, 1992). p53 content increases after alterations of DNA, blocking the cell cycle and allowing DNA repair, or leading to apoptosis of the damaged cells. Tumour cells with inactivated p53 have generally displayed a greater degree of resistance to DNA toxic agents, such as alkylating compounds or radiotherapy (Fan et al, 1994; Lowe et al, 1994). This resistance phenomenon can be explained by reduced sensitivity to apoptosis, and is often associated with gross genomic alterations including an aneuploid karyotype.

Alterations in p53 also abolish the $G_2$/M checkpoint and allow endoreduplication in the presence of TBA (Cross et al, 1995). It was therefore striking that the loss of wild-type p53 (wt-p53) function has been found by some authors to be associated with increased sensitivity to TBA, such as paclitaxel (Hawkins et al, 1996; Wahl et al, 1996). However other authors have reported that the loss of wt-p53 led to increased resistance to TBA (Wu and El-Diery, 1996) or did not modify sensitivity to these compounds (Delia et al, 1996). The present study was designed to investigate whether inactivation of wt-p53 protein by a dominant negative mutant affected sensitivity to the cytotoxicity of tubulin-binding agents and to determine the role of p53 in the pathways that link microtubule damage, mitotic arrest and cell death induced by these agents.

### MATERIALS AND METHODS

### Reagents

Drugs used for the experiments were clinical formulation of vinblastine, vincristine, vindesine (Lilly Laboratories, Saint Cloud, France), vinorelbine (Pierre Fabre, Castres, France), paclitaxel (Bristol-Myers Squibb, Paris, France), docetaxel (Rhône-Poulenc Rorer, Vitry-sur-Seine, France), mitomycin C (Sanofi Winthrop, Gentilly, France) and doxorubicin (Pharmacia, St Quentin, France). Flavopiridol was a kind gift from Hoescht Roussel pharmaceuticals (Paris, France). Stock solutions of each drug were prepared in distilled water or DMSO and fresh dilutions were prepared before each experiment. Colchine, 3-[4,5-dimethylthiazol-2-yl]-2,5-diphenyl-tetrazolium bromide (MTT) and propidium iodide were purchased from Sigma (St Quentin Fallavier, France). Enhanced chemiluminiscence Western blot detection reagents were purchased from Amersham Corp (Amersham ECL system, Buckinghamshire, UK). Antibodies against cyclin-G1, Bax and Raf-1 (C-12) were purchased from Santa Cruz Biotechnology Inc (Santa Cruz, CA); antibodies against Bcl-2 and p53 (DO7) were purchased from DAKO (Glostrup, Denmark); antibodies against (Poly[ADP-Ribose] Polymerase) (PARP) were purchased from Transduction Laboratories (Lexington, KY), Pharmigen (San Diego, CA) and Boehringer Mannheim (Gmbh, Germany), respectively. Peroxidase-conjugated secondary antibodies were purchased from Covalab (Oullins, France). Tetra methyl rhodamine methyl ester (TMRM) was purchased from Molecular probes (Interchim, Montluçon, France).

### Cell lines

The MN-1 cell line, containing wt-p53 and the MDD2 cell line, derived from the human breast carcinoma cell line MCF-7, were generously provided by Moshe Oren (Weizmann Institute of Science, Israel). The MDD2 line is a variant derived from MCF-7 by transfection with a dominant negative mutant of p53 (pCMV-DD-p53; mut-p53). This plasmid encodes a non-functional p53 miniprotein containing the first 11 residues and residues 302 to 390 of murine p53 (Shaulian et al, 1992). The MN-1 line is a control line transfected with the empty plasmid. HCT116+/+ and HCT116–/– are cell lines derived from colorectal carcinoma. HCT116+/+ displays wt-p53 while in HCT116–/–, 2 promoterless targeting vectors were used to sequentially disrupt the 2 p53 alleles (Bunz et al, 1998). All cell lines were maintained in monolayer cultures on 75 $cm^2$ flasks in Dulbecco's Minimum Essential Medium containing 10% fetal calf serum, 1% L-glutamine, 2% penicillin-streptomycin-neomycin (0.4 mg $ml^{-1}$).

### Cytotoxicity assays

Cell viability was determined using the MTT assay as previously described (Twentyman et al, 1989). The inhibitory concentration 50 ($IC_{50}$) was defined as the drug concentration resulting in 50% loss of cell viability relative to untreated cells. Assays were performed in triplicate in at least 3 separate experiments. In experiments with flavopiridol, this compound was added at a final concentration of 300 nM 6 hours after exposure to vinblastine or paclitaxel.

### Flow cytometry analyses

The apoptotic fraction was determined using the Annexin-V-Fluos Staining Kit (Boehringer Mannheim, Gmbh, Germany) after 24 h treatment of cells with vinblastine 200 nM or paclitaxel 100 nM. Cell cycle distribution and DNA ploidy status after 24 h of exposure to vinblastine or paclitaxel were calculated from propidium iodide-stained cells using Modfit LT 2.0™ software (Verity Software Inc, Topsham, ME). In experiments with flavopiridol, this compound was added at a final concentration of 300 nM 6 hours after exposure to vinblastine or paclitaxel and cell cycle distribution and DNA ploidy status determined after 24 h treatment.

### Western blots

Protein expression was determined by Western blot analysis in untreated MN-1 and MDD2 cells and after 24 h exposure to vinblastine (200 nM) or paclitaxel (100 nM) as previously described (Dumontet et al, 1996). Briefly, cell lysates were resolved by 12% SDS (sodium-dodecyl sulfate)-PAGE, and transferred onto nitro-cellulose membrane (Hybond-ECL, Amersham Corp, Buckinghamshire, UK). The blots were then incubated with the appropriate dilution of primary antibody, followed by incubation with peroxidase-conjugated secondary antibody. Protein were detected by chemiluminescence using Kodak film (Eastman Kodak Company, Rochester, NY, USA).

### Northern blots

Total RNA was extracted using Tri-Reagent (Sigma). This procedure is an improvement of the single-step method reported by Chomczynski and Sacchi for total RNA isolation (Chomczynski and Sacchi, 1987). RNA samples (10 µg) were separated by electrophoresis through a denaturing formaldehyde agarose gel and transferred tonylon membranes (Hybond-$N^+$; Amersham). Membranes were labelled with a p21/WAF1 cDNA probe.

### Measurement of mitochondrial transmembrane potential analysis

Control and TBA-treated cells were incubated in medium containing 500 nM (TMRM) for 15 min at 37˚C. Then, cells were distrupted with 1 ml Hanks buffer saline solution (HBSS) 0.25% SDS and TMRM fluorescence was measured at 574 nm after excitation at 546 nm using a spectrofluorophotometer RF5301PC. TMRM amounts were estimated using a TMRM calibration curve performed under the same conditions. The results are expressed as: Δ[TMRM]% = ([TMRM]assay – [TMRM]control) / [TMRM] control × 100.

### Assays for caspases 3 and 9

Determination of activation of caspase 3 and 9 at baseline and after 24 h of vinblastine and paclitaxel incubation were performed as previously described (Voorzanger-Rousselot et al, 1998).

### Determination of Raf-1 kinase activity

Raf-1 kinase activity was determined as described earlier (Rasouli-Nia et al, 1998). The level of Raf-1 kinase activity

© 2001 Cancer Research Campaign

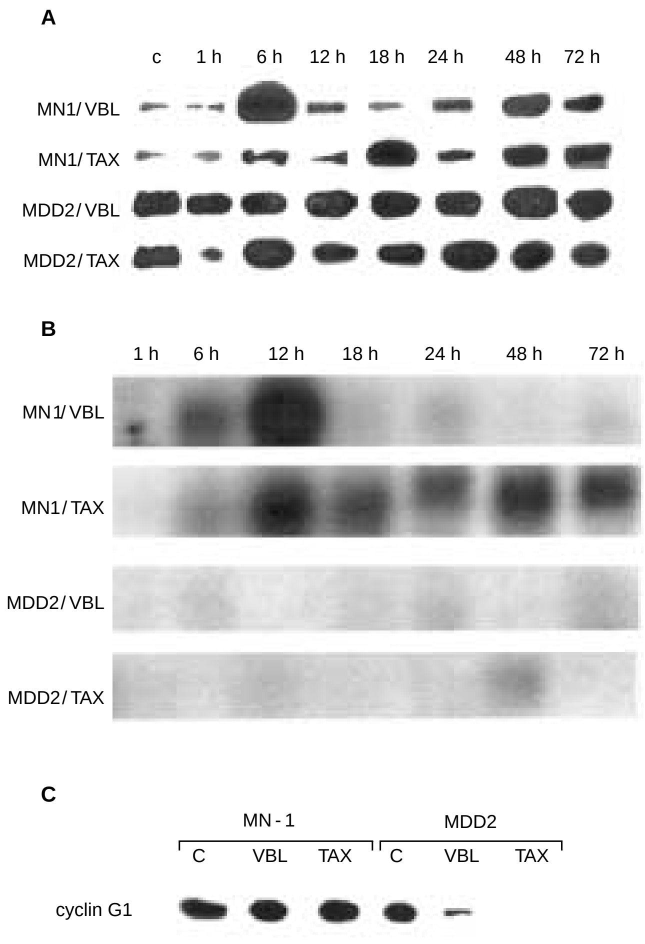

**Figure 1** Induction of p53 protein and p53-related events in MN-1 and MDD2 cells after exposure to 200 nM vinblastine (VBL) and 100 nM paclitaxel (TAX). (**A**) Kinetics of p53 protein. Western blots were performed on 50 µg of total cell protein with DO7 anti-p53 antibody; (**B**) p21/WAF1 RNA levels were determined by Northern blotting; (**C**) cyclin-G1 protein expression was measured by Western blot after 24 h exposure to both drugs; results shown are representative of 2 separate experiments was determined using the Pierce Colorimetric PKC assay kit Spinzyme™ Format (Pierce, Rockford, IL) according to the manufacturer's instructions and the fluorescence intensity determined by fluorescence spectroscopy.

### Electron microscopy

Control and treated cell lines were fixed in situ in sodium cacodylate 0.1 M buffer containing 2% glutaraldehyde at 4° for 2 h, washed in the same buffer and post-fixed in sodium cacodylate 0.15 M buffer containing 1% osmium tetroxide and 1.5% potassium ferrocyanide for 1 h. The cells were then dehydrated in graded ethanol and embedded in epoxy resin. Ultrathin sections were double-contrasted with uranyle acetate and lead citrate and examined at 80 KV on a Siemens Elmiskop 102 transmission electron microscope.

### RESULTS

### Induction of p53 protein by tubulin-binding agents p53 protein expression was determined after exposure to vinblastine and paclitaxel at different time intervals. In MN-1 cells, exposure to these drugs induced significant wt-p53 acumulation in a time-dependent manner with maximum effects after 6 h for vinblastine and 18 h for paclitaxel (Figure 1A). p53 accumulation could be detected up to 72 h with both compounds. In MDD2 cells, p53 was detected at high levels at all time points, since the mutant p53 transfected in these cells is recognized by the DO7 antibody (Figure 1A).

### Effect of p53 status on sensitivity to chemotherapeutic agents

$IC_{50}$ values of various chemotherapeutic compounds on MN1 and MDD2 cells are shown in Table 1. MDD2 cells displayed 31.5 to 50-fold resistance to paclitaxel and vinblastine in comparison to MN1 cells. In similar experiments with HCT-116+/+ (wt-p53) and

**Table 1** Cytotoxicity data (IC50)[a] and relative resistance ratio (RR)[b] values to different drugs

| Drug (nM) | MCF-7 | MN1 | MDD2 | RR | HCT +/+ | HCT –/– | RR |
|---|---|---|---|---|---|---|---|
| VBL[c] | 7.4 | 6.8 | 340 | 50 | 0.2 | 0.53 | 2.6 |
| NBL | ND | 8.2 | 23 | 2.8 | ND | ND | ND |
| VDS | ND | 6 | 10 000 | 1 666 | ND | ND | ND |
| VCR | ND | 58 | 3 400 | 58.6 | ND | ND | ND |
| TAX | 9 | 7.6 | 240 | 31.5 | 0.05 | 0.13 | 2.6 |
| TXT | ND | 8.7 | 194 | 22.2 | 0.5 | 1.5 | 3 |
| COL | ND | 85 | 185 000 | 2 176 | 160 | 950 | 5.9 |
| MITO | ND | 645 | 6 450 | 10 | 167 | 950 | 5.6 |
| DOX | 45 | 53 | 110 | 2.07 | 30 | 190 | 6.3 |

[a]IC50:IC50 values were obtained from dose response curves assessed by MTT assay and are means of three separate experiments each of which were performed in triplicate. SD were < 10%. [b]RR: relative resistance ratio (IC50 MDD2/IC50 MN1 or IC50 HCT116+/+/HCT116–/–). [c]VBL: vinblastine; NBL: vinorelbine; VDS: vindesine; VCR: vincristine; TAX: paclitaxel; TXT: docetaxel; COL: colchicine; MITO: mitomycin C; DOX: doxorubicin; ND: not determined.

 *© 2001 Cancer Research Campaign*

HCT-116–/– cells exposed to TBA, the survival advantage of cells carrying a non-functional p53 was confirmed, although to a lesser degree (Table 1). MN1 and MDD2 cells were negative for classical MDR, MRP and LRP resistance proteins by immunostaining and flow cytometric functional assays (data not shown).

### Influence of TBA on cell cycle arrest according to p53 status

Flow-cytometric cell cycle analysis demonstrated a diploid-MN-1 cell population that after 24 h of vinblastine treatment, accumulated in $G_2$/M phase (Table 2). Similar features were observed when MN-1 cells were exposed to paclitaxel (Table 2). Conversely, MDD2 cells demonstrated a partially tetraploid histogram (DI = 1.93 ± 0.02; $n$ = 3). In these cells, TBA treatment induced the presence of a large number of cells with 8N DNA content (Table 2). Thus, these results demonstrate that treatment of MN1 and MDD2 cells with TBA produces an accumulation of cells within $G_2$/M phase independently of the presence or not of a functional p53.

### Induction of p53-related events after exposure to vinblastine and paclitaxel

We examined whether these agents required a functional p53 to induce expression of different proteins involved in the $G_2$/M checkpoint. p21/WAF1 mRNA expression was analysed by Northern blotting and cyclin-$G_1$ expression by Western blotting. As shown in Figure 1B, p21/WAF1 expression was strongly induced in MN1 cells after 12 h of vinblastine or paclitaxel treatment. However, p21/WAF1 levels decreased 24 h after exposure to vinblastine and remained stable up to 72 hours after exposure to paclitaxel. In MDD2 cells, exposure to TBA treatment induced very weak expression of p21/WAF1 mRNA (Figure 1B). Exposure to vinblastine or paclitaxel for 24 h resulted in a moderate increase in cyclin-$G_1$ protein levels in MN-1 cells and in a decrease of this protein in MDD2 cells (Figure 1C). These results indicate that alterations in p21/WAF1 and cyclin-$G_1$ expression after treatment with tubulin-binding agents are p53-dependent.

**Table 2** Analysis of ploidy and cell cycle distribution of MN1 and MDD2 cells by flow cytometry before and after 24 h treatment with vinblastine and paclitaxel[a]

| | MN1 (wt-p53) | MDD2 (mut-p53) | |
|---|---|---|---|
| Cell population | D[b] (100) | D (68 ± 10.1) | T (32.2 ± 10) |
| G2/M CON[c] | 11.8 ± 5.1 | ND | 5.2 ± 7.3 |
| G2/M VBL | 68.4 ± 7.9 | ND | 52.7 ± 11.7 |
| G2/M TAX | 64.7 ± 6.4 | ND | 62.7 ± 10.6 |

[a]The reported values (mean ± SD, n = 3) are expressed as percentage of total cells. [b]D: diploid cells, 2N; T: tetraploid cells, 4N; ND: not detectable. [c]CON: control; VBL: vinblastine; TAX: paclitaxel; ND: not determined.

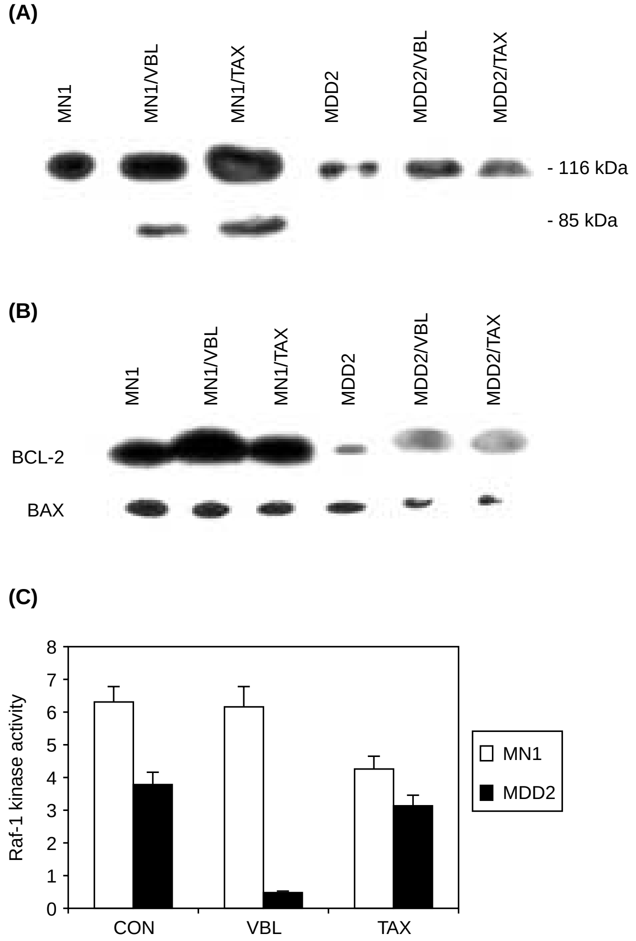

**Figure 2** Effect of antitubulin-binding agents on PARP cleavage (**A**), protein expression of Bcl-2 and Bax (**B**) and Raf-1 kinase activity (**C**) in MN-1 and MDD2 cells. Cells were treated with vinblastine 200 nM and paclitaxel 100 nM for 24 h. For (**A**) and (**B**), cells were lysed; subjected to SDS-PAGE, and immunoblotted with corresponding monoclonal antibody as described in Material and methods. For (**C**), Raf-1 kinase activity is expressed in IU/$10^6$ cells. This figure represents one of the two experiments performed. CON: control; VBL: vinblastine; TAX: paclitaxel

**Table 3** Apoptotic percentage, mitochondrial transmembrane potential variations and caspase 3 and 9 activity in MN1 and MDD2 cells exposed to tubulin-binding agents

| | Apoptosis[a] (%) | [TMRM][b] (nmol mg$^{-1}$) | △ [TMRM] (%) | Caspase 3 (pmol min$^{-1}$) | Caspase 9 (pmol min$^{-1}$) |
|---|---|---|---|---|---|
| MN-1 CON[c] | 2 | 0.146 | – | 36.7 | 31.0 |
| MN-1 VBL | 43 | 0.231 | + 58.2 | 29.3 | 27.5 |
| MN-1 TAX | 44 | 0.264 | + 80.8 | 30.4 | 26.7 |
| MDD2 CON | 3 | 0.145 | – | 33.1 | 28.1 |
| MDD2 VBL | 12 | 0.168 | + 11.7 | 28.7 | 21.7 |
| MDD2 TAX | 25 | 0.211 | + 45.5 | 32.8 | 29.1 |

[a]As measured by the flow cytometric annexin-V-Fluos Staining Kit. [b]TMRM: tetra methyl rhodamine methyl ester; The results are expressed as: △[TMRM]% = ([TMRM] assay – [TMRM]control) / [TMRM] control × 100.[c]CON:Control; VBL:vinblastine; TAX:paclitaxel.

*© 2001 Cancer Research Campaign* 

### Effect of p53 status on TBA-induced apoptosis

After exposure of the MN1 line to vinblastine for 24 h, 43% of cells were found to be apopotic in contrast to 12% of MDD2 cells (Table 3). Similar results were obtained after a 24 h exposure to paclitaxel (Table 3). Analysis of PARP cleavage was detected after vinblastine and paclitaxel treatment in MN1 cells but not in MDD2 cells (Figure 2A).

### Effect of TBA on pro- and anti-apoptotic proteins

Expression of Bcl-2 and Bax proteins were examined in MN1 and MDD2 cells after exposure to TBA. At baseline, MN1 cells had significantly higher Bcl-2 and Bax expression than MDD2 cells (Figure 2B). Bcl-2 protein increased after drug exposure in both cell lines. Bax protein level was reduced in the MDD2 line after exposure to vinblastine or paclitaxel but remained stable in the MN1 line after exposure to these compounds.

### Effect of exposure to TBA on transmembrane mitochondrial potential and caspase 3 and 9 activity

To investigate the mitochondrial changes that precede apoptosis, we measured the mitochondrial transmembrane potential in untreated and treated cells using the accumulation of TMRM. The increase in TMRM accumulation ratio after drug treatment was weaker in the mut-p53 cells than in the wt-p53 cells (Table 3). caspase 3 and 9 activities were similar in both cell lines, and no increase in caspase 3 or 9 activity was observed in either line after exposure to vinblastine or paclitaxel (Table 3), suggesting that PARP cleavage may be due to the activity of other caspases.

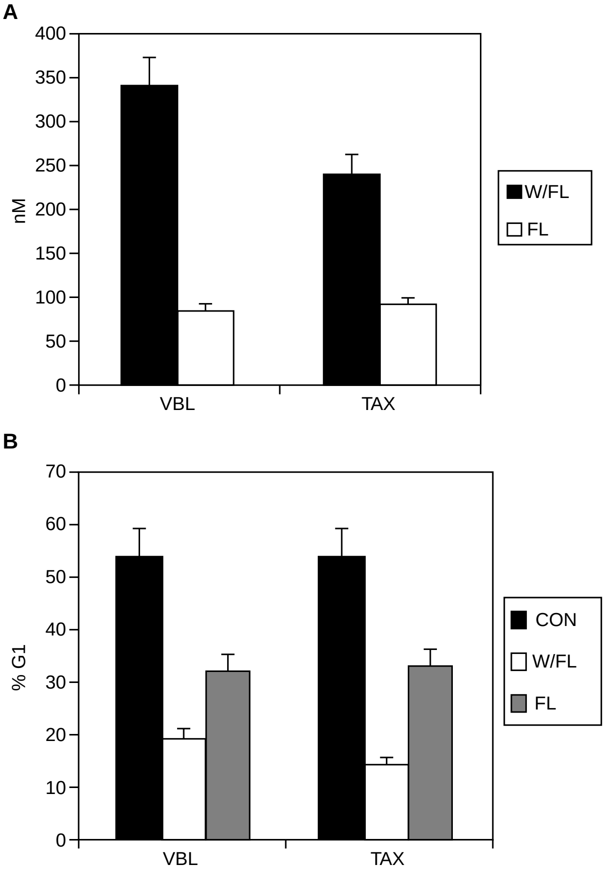

**Figure 3** Effect of sequential treatment with vinblastine (VBL) or paclitaxel (TAX) and flavopiridol (FL) on MDD2 cells. (**A**) $IC_{50}$ values; MDD2 cells were plated at $7 \times 10^3$ cells/well in the presence of increasing concentrations of vinblastine (VBL) and paclitaxel (TAX). After 6 h of incubation, flavopiridol (FL) 300 nM was added. Cytotoxic data was determined by the MTT assay after 72-h incubation; (**B**) Percentage of cells in G1 phase; FL 300 nM was added to 6-h VBL- and TAX-treated MDD2 cells and incubated for 24 h. Cell cycle distribution was analysed by flow cytometry. CON: control; W/FL: without flavopiridol; Add FL: flavopiridol-treated MDD2 cells

### Effect of exposure to TBA on Raf-1 kinase activity

At baseline, MN1 cells had a 2-fold greater Raf-1 kinase activity than MDD2 cells (Figure 2C). Exposure of MN1 cells to vinblastine did not change the level of activity of Raf-1 kinase, while exposure to paclitaxel reduced Raf-1 activity to 68% of the baseline value. Conversely, vinblastine exposure of MDD2 cells reduced Raf-1 kinase activity 8.6-fold, while exposure to paclitaxel had no effect. We observed no difference in Raf-1 protein expression between the 2 cell lines, either before or after treatment with vinblastine or paclitaxel (data not shown).

### Effect of exposure to TBA on microtubular organization

The ultrastructural analysis showed that mut-p53 MDD2 cells but not wt-p53 MN1 cells displayed atypical structures in the presence of drugs. MDD2 exposed to vinblastine exhibited lamellar complexes studded with ribosomes (data not shown). These structures were similar to the so-called ribosome–lamella complexes. When exposed to paclitaxel, MDD2 showed long tubulo-filamentous structures (data not shown). MN1 cells did not display similar structures after drug exposure.

### Effect of flavopiridol on the chemoresistant phenotype of MDD2 cells

There were significant differences between $IC_{50}$ values of MDD2 cells treated with vinblastine alone (340 nM) or with flavopiridol (85 nM) (Figure 3A). Similar results were found after MDD2 treatment with paclitaxel alone (240 nM) or with flavopiridol (90 nM) (Figure 3A). The addition of flavopiridol thus effectively reversed the resistant phenotype of MDD2 cells. Analysis of cell cycle alterations after sequential exposure to TBA and flavopiridol showed $G_1$ arrest in this cell line. Figure 3B shows that after vinblastine and paclitaxel treatment, the amount of cells in $G_1$ phase diminished as a result of the $G_2$/M arrest caused by these drugs. Sequential treatment with vinblastine or paclitaxel followed by flavopiridol increased the amount of cells in $G_1$ phase.

### DISCUSSION

The effect of loss of p53 function on sensitivity to tubulin-binding agents has yielded conflicting results. Wu and co-workers found that p53 status predicted in vitro chemoresistance to paclitaxel in PA-1 human ovarian teratocarcinoma cells that expressed HPV16 E6 protein (Wu and El-Diery, 1996). In contrast, Whal et al and Hawkins et al found that cells became more sensitive to paclitaxel if they lacked p53 (Hawkins et al, 1996; Wahl et al, 1996) and increased sensitivity to paclitaxel correlated with increased $G_2$/M cell cycle arrest and apoptosis induction in cells lacking functional p53. These studies used human foreskin fibroblasts and mouse embryo fibroblasts, respectively, in which p53 inactivation was achieved either

 © 2001 Cancer Research Campaign through targeted disruption of the gene or functional inactivation by acute expression of HPV16 E6 or SV40 T antigen. Such cells are more sensitive to apoptosis induced by chemotherapy, hypoxia or growth factor withdrawal than cancer cells (McGill et al, 1997; Brown and Wouters, 1999). Conversely, Delia and co-workers demonstrated that the sensitivity to paclitaxel was similar in EBV immortalized lymphoblastoid cells carrying heterozygous mutations of p53 (p53 wt/mut) or wt-p53 (wt/wt) (Delia et al, 1996). However, Brown and Wouters (Brown and Wouters, 1999) reported that, similarly to oncogene-transformed normal cells, apoptotic characteristics of lymphoid cells are different from those of cells derived from solid tumours.

Our results obtained with MCF-7 variants show that inactivation of wt-p53 by a dominant negative mutant p53 miniprotein is associated with a high degree of resistance to tubulin-binding agents. Similar results were obtained with the colon-derived line HCT116 p53 +/+ and –/–, although to a lesser degree. It is therefore possible that p53 alterations do not have the same consequences in terms of chemosensitivity in cells transformed in vitro and in tumour-derived cancer cells. In normal cells with inactivated p53, the absence of cell cycle arrest mechanisms allowing adequate chromosome distribution during anaphase may predominate, explaining increased propensity to apoptosis and enhanced sensitivity to chemotherapy. Conversely in neoplastic cells which have already successfully survived aberrant chromosomal distribution in vivo, the absence of the apoptosis-inducing activity of p53 may represent a survival advantage. It must be stressed that the use of lines stably transfected with dominant negative p53 variants has a major caveat which is the genetic drift over time with accumulation of chromosomal aberrations. The mut-p53 line used in this study is partially tetraploid, with a certain number of chromosomal aberrations which may be involved in chemoresistance mechanisms. One way to avoid this problem is to study inducible p53 variants, an approach which is currently being developed in our laboratory.

In our model, p53 status did not influence the ability of cells to accumulate in $G_2$/M. However wt-p53 cells were essentially found to be blocked in the 4N state after drug treatment, whereas the mut-p53 line, which is partially tetraploid, accumulated with an 8N DNA content, suggesting that the absence of p53 allowed endoreduplication. Other authors have confirmed that loss of p53 leads to microtubule-induced endoreduplication and generation of 8N cell populations (Cross et al, 1995; Khan and Wahl, 1998). Cyclin $G_1$ levels, which are involved in molecular events mediating $G_2$/M transition (Bates et al, 1996) were identical at baseline in both cell lines suggesting that expression of this protein is at least partially p53-independent. Cyclin $G_1$ levels increased only moderately in the wt-p53 cells exposed to TBA in spite of p53 protein accumulation but were dramatically reduced in the mut-p53 cells. These data suggest that under situations of stress inducing a $G_2$/M cell block, these different events may favour endoreplication of mut-p53 cells.

Paclitaxel-induced apoptosis has been suggested to be mediated by Raf-1 kinase-mediated phosphorylation of Bcl-2 with concurrent loss of its anti-apoptotic properties (Blagosklonny et al, 1996; Haldar et al, 1998). In our model mut-p53 cells had lower levels of Raf-1 kinase activity than wt-p53 cells. This is in keeping with the recent report (Fang et al, 2000) showing that wt-p53 expression induces an activation of the MAPK cascade by a mechanism which is upstream of Ras. Raf-1 kinase activity was slightly decreased in both lines after exposure to paclitaxel. Exposure to vinblastine induced a 90% decrease in Raf-1 kinase activity in mut-p53 cells and only a 30% decrease in wt-p53 cells. It has been reported that the effect of TBA on Raf-1 kinase requires an effect on tubulin polymerization (Blagosklonny et al, 1996). Electron microscopy studies showed that mut-p53 MDD2 cells, but not wt-p53 cells, displayed abnormal microtubular reorganization induced by these drugs. The effects of paclitaxel and vinblastine on Raf-1 kinase in the mut-p53 line may therefore be due to microtubular reorganization induced by TBA.

Paclitaxel-induced apoptosis has been reported to be associated with changes in mitochondrial transmembrane potential, mitochondrial release of cytochrome c, activation of caspase 3 and cleavage of PARP (Ibrado et al, 1998; Scarlett et al, 2000). Our results show strongly reduced Bcl-2 levels and only weakly reduced Bax levels in the mut-p53 cells in comparison to wt-p53 cells. It is therefore likely that the Bcl-2:Bax ratio at baseline is higher in MN1 cells than in MDD2 cells. After drug treatment, there may occur an increase in the Bcl-2:Bax ratio in the mut-p53 cell line while the Bcl-2:Bax ratio in the wt-p53 cells remains stable, conferring a survival advantage to mut-p53 MDD2 cells. Analysis of mitochondrial transmembrane potential showed a weaker variation in the mut-p53 cells than in wt-p53 cells, suggesting a reduced release of cytochrome c from mitochondria. It is presently uncertain whether loss of mitochondrial membrane potential represents the central initiating event in apoptosis (Yang et al, 1997; Bossy-Wetzel et al, 1998). Caspase 3 and 9 levels were identical in both cells lines, and no increase in caspase 3 or 9 activity was observed in either line after exposure to TBA. Other activators of PARP, such as caspase 7 may be involved in PARP activation. Taken together these data suggest that TBA-induced apoptosis involves a mitochondrial process, that this process is partially p53-dependent, but does not involve caspase 3.

Flavopiridol, a synthetic flavone presently undergoing phase II clinical trials, prevents endoreduplication in human cancer cells defective in $G_1$ checkpoint proteins (Motwani et al, 2000). The effect of flavopiridol on cell cycle arrest has been reported to be independent of p53 status (Byrd et al, 1998). In our model sequential exposure to TBA and flavopiridol effectively reversed the drug-resistant phenotype of mut-p53 cells. When analysing cell cycle perturbations induced by the sequential treatment, addition of flavopiridol increased the amount of cells in $G_1$ peak indicating that the additive effect of flavopiridol and vinblastine or paclitaxel may be produced by preventing endoreplication in cells with a compromised $G_1$ checkpoint. Although p53-deficient cells ignore the $G_2$/M checkpoint and progress into $G_1$ in spite of microtubular insult, it is thus possible to block this progression pharmacologically and sensitize mut-p53 cells to TBA cytoxicity.

### ACKNOWLEDGEMENTS

Carlos Galmarini is a recipient of the 'Michel Clavel' grant. This study was supported in part by the Ligue Contre le Cancer de l'Ardèche and the Association pour la Recherche contre le Cancer (ARC).

### REFERENCES

Bates S, Rowan S and Vousden KH (1996) Characterisation of human cyclin G1 and G2: DNA damage inducible genes. *Oncogene* **13**: 1103–1109

Blagosklonny MV, Schulte T, Nguyen P, Trepel J and Neckers LM (1996) Taxol-induced apoptosis and phosphorylation of Bcl-2 protein involves c-Raf-1 and represents a novel c-Raf-1 signal transduction pathway. *Cancer Res* **56**: 1851–1854

© 2001 Cancer Research Campaign 

Bossy-Wetzel E, Newmeyer DD and Green DR (1998) Mitochondrial cytochrome c release in apoptosis occurs upstream of DEVD-specific caspase activation and independently of mitochondrial transmembrane depolarization. *EMBO J* **17**: 37–49

Brown JM and Wouters BG (1999) Apoptosis, p53, and tumor cell sensitivity to anticancer agents. *Cancer Res* **59**: 1391–1399

Bunz F, Dutriaux A, Lengauer C, Waldman T, Zhou S, Brown JP, Sedivy JM, Kinzler KW and Vogelstein B (1998) Requirement for p53 and p21 to sustain G2 arrest after DNA damage. *Science* **282**: 1497–1501

Byrd JC, Shinn C, Waselenko JK, Fuchs EJ, Lehman TA, Nguyen PL, Flinn IW, Diehl LF, Sausville E and Grever MR (1998) Flavopiridol induces apoptosis in chronic lymphocytic leukemia cells via activation of caspase-3 without evidence of bcl-2 modulation or dependence on functional p53. *Blood* **92**: 3804–3816

Chomczynski P and Sacchi N (1987) Single-step method of RNA isolation by acid guanidinium thiocyanate-phenol-chloroform extraction. *Anal Biochem* **162**: 156–159

Cross SM, Sanchez CA, Morgan CA, Schimke MK, Ramel S, Idzerda RL, Raskind WH and Reid BJ (1995) A p53-dependent mouse spindle checkpoint. *Science* **267**: 1353–1356

Delia D, Mizutani S, Lamorte G, Goi K, Iwata S and Pierotti MA (1996) p53 activity and chemotherapy. *Nat Med* **2**: 724–725

Dumontet C, Duran GE, Steger KA, Beketic-Oreskovic L and Sikic BI (1996) Resistance mechanisms in human sarcoma mutants derived by single-step exposure to paclitaxel (Taxol). *Cancer Res* **56**: 1091–1097

Fan S, el-Deiry WS, Bae I, Freeman J, Jondle D, Bhatia K, Fornace AJ, Jr., Magrath I, Kohn KW and O'Connor PM (1994) p53 gene mutations are associated with decreased sensitivity of human lymphoma cells to DNA damaging agents. *Cancer Res* **54**: 5824–5830

Fang L, Lee S, Igarashi M, Ouchi T, Lu K and Aaronson A (2000) Cross-talk between the tumor suppressor p53 and the Ras/Raf/MAPK cascade. *Proc 10th Int p53 Workshop* **10**: 100A

Haldar S, Basu A and Croce CM (1998) Serine-70 is one of the critical sites for drug-induced Bcl2 phosphorylation in cancer cells. *Cancer Res* **58**: 1609–1615

Hawkins DS, Demers GW and Galloway DA (1996) Inactivation of p53 enhances sensitivity to multiple chemotherapeutic agents. *Cancer Res* **56**: 892–898

Ibrado AM, Kim CN and Bhalla K (1998) Temporal relationship of CDK1 activation and mitotic arrest to cytosolic accumulation of cytochrome C and caspase-3 activity during Taxol-induced apoptosis of human AML HL-60 cells. *Leukemia* **12**: 1930–1936

Jordan MA, Thrower D and Wilson L (1992) Effects of vinblastine, podophyllotoxin and nocodazole on mitotic spindles. Implications for the role of microtubule dynamics in mitosis. *J Cell Sci* **102**: 401–416

Jordan MA, Toso RJ, Thrower D and Wilson L (1993) Mechanism of mitotic block and inhibition of cell proliferation by taxol at low concentrations. *Proc Natl Acad Sci USA* **90**: 9552–9556

Jordan MA, Wendell K, Gardiner S, Derry WB, Copp H and Wilson L (1996) Mitotic block induced in HeLa cells by low concentrations of paclitaxel (Taxol) results in abnormal mitotic exit and apoptotic cell death. *Cancer Res* **56**: 816–825

Khan SH and Wahl GM (1998) p53 and pRb prevent rereplication in response to microtubule inhibitors by mediating a reversible G1 arrest. *Cancer Res* **58**: 396–401

Lane DP (1992) Cancer. p53, guardian of the genome. *Nature* **358**: 15–16

Liu Y, Bhalla K, Hill C and Priest DG (1994) Evidence for involvement of tyrosine phosphorylation in taxol-induced apoptosis in a human ovarian tumor cell line. *Biochem Pharmacol* **48**: 1265–1272

Lobert S, Vulevic B and Correia JJ (1996) Interaction of vinca alkaloids with tubulin: a comparison of vinblastine, vincristine, and vinorelbine. *Biochemistry* **35**: 6806–6814

Lowe SW, Bodis S, McClatchey A, Remington L, Ruley HE, Fisher DE, Housman DE and Jacks T (1994) p53 status and the efficacy of cancer therapy in vivo. *Science* **266**: 807–810

McGill G, Shimamura A, Bates RC, Savage RE and Fisher DE (1997) Loss of matrix adhesion triggers rapid transformation-selective apoptosis in fibroblasts. *J Cell Biol* **138**: 901–911

Motwani M, Li X and Schwartz GK (2000) Flavopiridol, a cyclin-dependent kinase inhibitor, prevents spindle inhibitor-induced endoreduplication in human cancer cells. *Clin Cancer Res* **6**: 924–932

Ngan VK, Bellman K, Panda D, Hill BT, Jordan MA and Wilson L (2000) Novel actions of the antitumor drugs vinflunine and vinorelbine on microtubules. *Cancer Res* **60**: 5045–5051

Paoletti A, Giocanti N, Favaudon V and Bornens M (1997) Pulse treatment of interphasic HeLa cells with nanomolar doses of docetaxel affects centrosome organization and leads to catastrophic exit of mitosis. *J Cell Sci* **110**: 2403–2415

Rasouli-Nia A, Liu D, Perdue S and Britten RA (1998) High Raf-1 kinase activity protects human tumor cells against paclitaxel-induced cytotoxicity. *Clin Cancer Res* **4**: 1111–1116

Rudner AD and Murray AW (1996) The spindle assembly checkpoint. *Curr Opin Cell Biol* **8**: 773–780

Scarlett JL, Sheard PW, Hughes G, Ledgerwood EC, Ku HH and Murphy MP (2000) Changes in mitochondrial membrane potential during staurosporine-induced apoptosis in Jurkat cells. *FEBS Lett* **475**: 267–272

Shaulian E, Zauberman A, Ginsberg D and Oren M (1992) Identification of a minimal transforming domain of p53: negative dominance through abrogation of sequence-specific DNA binding. *Mol Cell Biol* **12**: 5581–5592

Trielli MO, Andreassen PR, Lacroix FB and Margolis RL (1996) Differential Taxol-dependent arrest of transformed and nontransformed cells in the G1 phase of the cell cycle, and specific-related mortality of transformed cells. *J Cell Biol* **135**: 689–700

Twentyman PR, Fox NE and Rees JK (1989) Chemosensitivity testing of fresh leukaemia cells using the MTT colorimetric assay. *Br J Haematol* **71**: 19–24

Voorzanger-Rousselot N, Favrot M and Blay JY (1998) Resistance to cytotoxic chemotherapy induced by CD40 ligand in lymphoma cells. *Blood* **92**: 3381–3387

Wahl AF, Donaldson KL, Fairchild C, Lee FY, Foster SA, Demers GW and Galloway DA (1996) Loss of normal p53 function confers sensitization to Taxol by increasing G2/M arrest and apoptosis. *Nat Med* **2**: 72–79

Wu GS and El-Diery WS (1996) p53 and chemosensitivity. *Nat Med* **2**: 255–256

Yang J, Liu X, Bhalla K, Kim CN, Ibrado AM, Cai J, Peng TI, Jones DP and Wang X (1997) Prevention of apoptosis by Bcl-2: release of cytochrome c from mitochondria blocked. *Science* **275**: 1129–1132

 *© 2001 Cancer Research Campaign*
